# Primary Care Clinician Preferences and Perspectives on Multi-Cancer Detection Testing Across an Integrated Healthcare System

**DOI:** 10.3390/jpm15100452

**Published:** 2025-09-28

**Authors:** Jessica D. Austin, Ilyse A. Nelson, Jon C. Tilburt, Eric R. Ellinghysen, Claire Yee, Jaxon Quillen, Brian M. Dougan, John R. Presutti, Ryan T. Hurt, Niloy Jewel Samadder, Karthik Ghosh, Steven W. Ressler

**Affiliations:** 1 Division of Epidemiology, Department of Quantitative Health Sciences, Mayo Clinic, Phoenix, AZ 85054, USA; 2 Mayo Clinic Comprehensive Cancer Center, Mayo Clinic, Phoenix, AZ 85054, USA; nelson.ilyse@mayo.edu (I.A.N.); samadder.jewel@mayo.edu (N.J.S.); 3 Division of Biomedical Ethics, Department of Quantitative Health Sciences, Mayo Clinic, Phoenix, AZ 85054, USA; tilburt.jon@mayo.edu; 4 General Internal Medicine, Department of Medicine, Mayo Clinic, Phoenix, AZ 85054, USA; 5 Office of Information Security, Risk Department, Mayo Clinic, Rochester, MN 55905, USA; ellinghysen.eric@mayo.edu; 6 Division of Biostatistics, Department of Quantitative Health Sciences, Mayo Clinic, Phoenix, AZ 85054, USA; yee.claire@mayo.edu (C.Y.); quillen.jaxon@mayo.edu (J.Q.); 7 Department of Medicine, General Internal Medicine, Mayo Clinic, Rochester, MN 55905, USA; dougan.brian@mayo.edu (B.M.D.); hurt.ryan@mayo.edu (R.T.H.); ghosh.karthik@mayo.edu (K.G.); 8 Family Medicine, Department of Medicine, Mayo Clinic, Jacksonville, FL 32224, USA; presutti.john@mayo.edu; 9 Center for Individualized Medicine, Mayo Clinic, Jacksonville, FL 32224, USA; 10 Department of Medicine, Gastroenterology & Hepatology, Mayo Clinic, Phoenix, AZ 85054, USA; 11 Clinical Genomics, Mayo Clinic, Phoenix, AZ 85054, USA

**Keywords:** multicancer detection testing, primary care, perceptions, preferences, cancer screening

## Abstract

**Background/Objectives:** Multi-cancer detection (MCD) tests have emerged as a promising tool to redefine the landscape of early cancer detection. Implementation of this novel technology will likely fall to primary care clinicians (PCC). The purpose of this study is to characterize and explore differences in PCCs perceptions and preferences towards MCD testing. **Methods:** Between March and May of 2023, this cross-sectional survey was administered to 281 PCCs, including physicians and advanced care providers practicing within an integrated healthcare system spanning five states. The survey collected data on self-reported characteristics, perceptions of MCD testing, and preferences for learning about MCD testing. Analysis was limited to those with no prior experience with MCD testing (*N* = 181, response rate 22.8%). Descriptive statistics summarized key variables and chi-square tests assessed differences in perceptions and preferences by key characteristics. **Results:** Most PCCs were interested in MCD testing (66.3%), but limited knowledge/awareness of MCD testing and confidence to manage patients with a positive test were observed, along with concerns around cost (76.7%) and misuse/poor implementation. The primary preferences for learning about MCD testing were online courses or classroom instruction (64.5%). Significant differences in perceptions and preferences for learning were observed by location, degree, and years in practice. **Conclusions:** PCCs in our study held positive views towards MCD testing, but gaps and variation in knowledge and confidence towards MCD testing and concerns around the cost and misuse/poor implementation were observed. While efforts to train and educate all PCCs on MCD testing is a critical first step, more research is needed to understand how best to support implementation tailored to individual and system-level needs and characteristics.

## 1. Introduction

Cancer remains a leading cause of death in the United States (U.S.). Early detection often saves lives, but single-organ screening has relied on disparate screening modalities and preparations that hinder workflow integration, reduce scheduling efficiency, compromise compliance, and increase logistical costs [1]. Among cancers with guideline recommended standard of care screening procedures [2,3,4,5], concerns around under- and over-utilization, testing availability, and impact on healthcare system costs persist [1,6,7,8]. Moreover, nearly 60% of all cancer deaths occur among cancers for which there is no validated screening modality [9]. Novel, accurate, minimally invasive, and cost-effective strategies could improve early cancer detection of multiple cancers.

Blood-based multicancer detection (MCD) testing, a so-called “liquid biopsy,” has emerged as a promising tool poised to redefine the current cancer landscape [10,11]. MCD tests use biological samples such as blood, urine, saliva, or other bodily fluids to detect minute quantities of circulating biomarkers including cell-free tumor DNA, RNA, and proteins, providing a near universal, minimally invasive method to screen for multiple cancer types at point of care [10]. Numerous MCD screening tests are currently under development and/or undergoing clinical trials to test their safety and efficacy, with some claiming to detect 3 to 50 different cancers [12,13]. Findings from two prospective screening studies have been reported. The PATHFINDER study examined Galleri^®^ by GRAIL that interrogates cell-free non-coding sequences in peripheral blood to measure the extent and location of genomic DNA methylation patterns. Data from 6662 participants who were 50 years or older and with or without cancer from seven healthcare systems in the United States demonstrated a specificity of 99%, and sensitivity was 70% for 12 prespecified cancer types, with increasing sensitivity for higher stage disease [14]. The DETECT-A study evaluated CancerSEEK™ by Exact Sciences—a multi-analyte blood test incorporating DNA and protein biomarkers. The study enrolled 10,006 women ages 65–75 with no known cancer diagnosis and detected 26 individual cancers across all four stages with a sensitivity of 27.1%, a specificity of 98.9%, and a false positive rate of 1%—paving the way for further developmental studies and Exact Sciences’ next generation genomic-based MCD test, Cancerguard™ [12]. In addition to performance trials, economic studies suggest that MCD testing may be a cost-effective strategy for detecting cancer, especially for those with no guideline-recommended screening test that tend to be diagnosed at later stages, resulting in higher treatment costs [15,16].

Converse to current screening programs, the goal of MCD testing is to reduce cancer mortality across multiple cancers, particularly those without recommended screening tests. Modeling studies have suggested significant improvements in early cancer detection that could translate to better outcomes and lower healthcare costs [17]. Moreover, a blood test may be more feasible for low-resourced settings to implement due to low upfront costs, thereby increasing access to populations that are most susceptible to disparities [18]. Reductions in cancer mortality, however, will depend on the sensitivity of MCD tests for detecting cancers at an earlier stage, adherence to regular screening, and adherence to emerging diagnostic pathways and follow-up of care [19,20]. MCD tests are not currently approved by the Food and Drug Administration or endorsed by any clinical practice guidelines due to limited evidence supporting MCD safety and efficacy [21]. While evidence continues to emerge [10,13,14], MCD tests are already commercially available through the Clinical Laboratory Improvement Act alongside routine screening and being implemented with a physician referral and out-of-pocket cost to patients, raising critical questions around how best to implement MCD into patient care.

The clinical implementation of MCD testing will likely fall on primary care clinicians (PCCs), including physicians and advanced practice providers (APPs), who manage most cancer screening protocols in the U.S. As such, the perspective of PCCs toward MCD testing is central to informing future implementation efforts. Emerging evidence in this area suggests that healthcare clinicians, including PCCs, are largely receptive to MCD testing but note concerns around managing false positives, false negatives, time constraints, and cost burdens [22,23,24]. More studies are needed across diverse settings to confirm these findings. Additionally, few, if any, studies have explored differences in perceptions by individual (degree, specialty) and system level (location) factors or have characterized preferences for learning about MCD testing which is central to implementation. To address this gap, this study aims to characterize and explore differences in PCCs perceptions and preferences towards MCD testing.

## 2. Materials and Methods

We utilized a cross-sectional survey of PCCs, including physicians (MD and DO) and APPs (NP and PAs) within the Mayo Clinic Enterprise, which includes a community-based healthcare system (HCS) and three tertiary academic medical centers (TMCs) across five states. The study was conducted between March and May of 2024 and was deemed exempt from review by the Mayo Clinic Institutional Review Board.

### 2.1. Survey Development and Deployment

To develop the survey, an initial list of proposed questions was compiled by members of the project team, including clinician stakeholders. This group validated the survey content and strength of questions via pilot testing with 14 clinicians identified by operations leadership over a 2 week period, gathering feedback on clarity, comprehension, relevance, appropriateness, engagement, and overall experience. A total of 10 clinicians completed the pilot (71.4% response rate). The project team reviewed feedback and revised survey items. The final survey was administered to 1013 PCCs, identified via distribution lists obtained from operations leadership across all sites. The survey was administered using a phased approach, by site, with each site having 3 weeks to respond to the survey. Participants received an email detailing the purpose of the survey with an anonymous link and QR code. Reminders were sent weekly for 3 weeks before the survey period closed.

Final survey items (see Supplementary Material) ascertained self-reported characteristics including department/division (internal medicine, family medicine, women’s health, international, and other), practice location (TMC vs. HCS), degree (APP vs. MD/DO), years in practice (<10 years vs. ≥10 years), and prior experience with MCD testing. PCCs were also asked to indicate their level of agreement on a five-point Likert-type scale (0 = Strongly Disagree to 5 = Strongly Agree) to items designed to characterize perceptions towards MCD testing, including knowledge and awareness of MCD tests, interest and attitudes towards MCD testing, and confidence (self-efficacy) in one’s ability to interpret, communicate, and provide direct care to patients with a positive MCD test. Response options were dichotomized as strongly agree/somewhat agree vs. neither agree nor disagree/somewhat disagree/strongly disagree. PCCs were asked to select all that apply to concerns about the use of MCD testing in healthcare more broadly with an open-ended response option. Additionally, PCCs indicated preferences for learning about MCD testing on a five-point Likert-type scale (1 = do not prefer to 5 = prefer a great deal) with responses categorized as prefer a great deal/a lot vs. prefer a moderate amount vs. prefer slightly/do not prefer.

### 2.2. Data Analysis

A total of 231 PCCs completed the survey (response rate = 22.8%). This analysis was limited to those with no prior experience with MCD testing resulting in a final analytic sample of 181 respondents. Survey items assessing perceptions, concerns, and preferences for learning about MCD testing were summarized using frequencies with percentages, and chi-square tests were used to assess differences by location (TMC vs. HCS), degree (APP vs. physician), and years in practice (>10 years vs. ≤10 years). All calculations were performed using R with a *p*-value of 0.05 as statistically significant.

## 3. Results

Table 1 summarizes respondent characteristics overall. Among 181 PCCs included in the analysis, 59.1% worked at a TMC, 59.1% were clinicians, 61.9% worked in family medicine, and 63% reported being in practice for 10 years or more.

### 3.1. Perceptions Towards MCD Testing

Table 2 describes the knowledge, attitudes, and self-efficacy of PCCs towards MCD testing. Overall, 39% of all respondents were familiar with the basic concepts of MCD testing and only 35% reported that they understood how MCD testing works. While 66.3% were interested in using MCD tests in their practice and 67.8% indicated that MCD tests will take on a significant role in healthcare, 77.9% of PCCs were concerned about having time to explain the test to their patients, and 83.2% were worried about managing their patients with a positive result. Only 22.9% of PCCs felt confident in their ability to offer or provide direct care to patients based on MCD results and fewer (16.9%) felt confident in their ability to interpret and communicate MCD results. Less than half of all PCCs (41.2%) felt comfortable understanding the limitations of MCD tests.

Table 3 shows differences in perceptions by location, degree, and years in practice. Compared to TMCs, PCCs from HCSs reported significantly lower levels of agreement to being familiar with basic concepts of MCD (25% vs. 48.6%, *p* < 0.01) and understanding of how MCD testing works (22.2% vs. 43.8%, *p* < 0.01). We did not observe significant differences in attitudes or self-efficacy by location. Examining differences by degree, compared to physicians, APPs reported significantly lower levels of agreement to the following items: (1) I am familiar with basic concepts of MCD, (2) I understand how MCD testing works, (3) I feel confident in my ability to interpret and communicate MCD results to the patients, and (4) I am comfortable understanding the limitations of MCD tests. Additionally, those who had been in practice for at least 10 years or more reported significantly higher levels of agreement with being familiar with the basic concepts of MCD testing (44% vs. 29.2%, *p* = 0.04) and understanding how MCD testing works compared to those with less than 10 years in their role (42% vs. 23.1%, *p* = 0.01).

### 3.2. Concerns About MCD Tests in Healthcare

**Figure 1:**
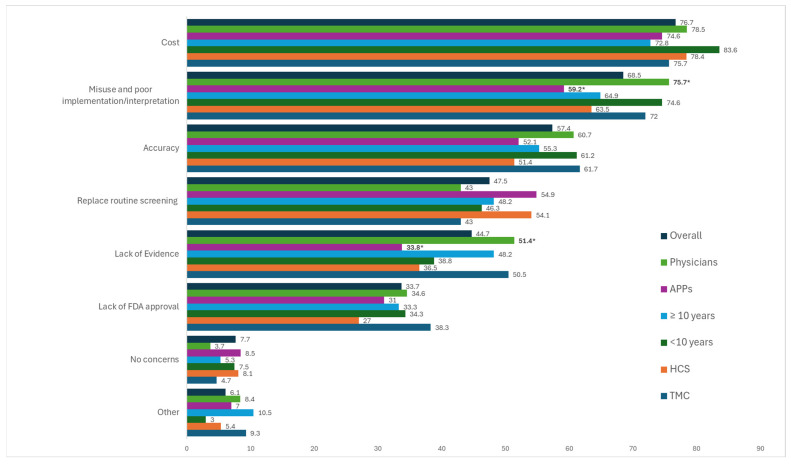
Concerns about MCD Tests in Healthcare Overall and by Location, Degree, and Years in Practice. *, Indicates statistically significant differences. *p*-value < 0.05.

Per Figure 1, cost (76.7%), misuse and poor implementation/interpretation (68.5%), and accuracy of test (57.4%) were reported as the top concerns overall, followed by concerns around MCD tests replacing routine screening (47.5%), lack of trial evidence (44.7%), and lack of FDA approval (33.7%).

**Figure 2:**
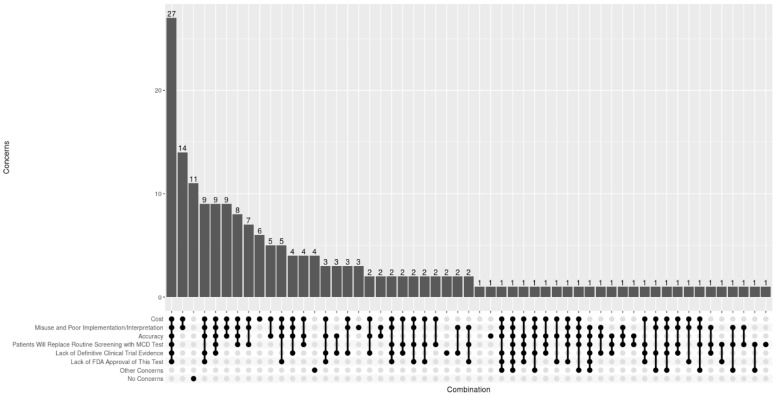
Boxplot summarizing combination of concerns about MCD tests in healthcare endorsed for all PCCs. The black dots indicate selected options.

Examining the combination of responses from all respondents (Figure 2), there were 54 unique combinations with 60.2% (*N* = 109) of respondents selecting both cost and misuse/poor implementation as a concern. Regarding differences in concerns by location, degree, and years in practice (see Figure 1), MD/DOs endorsed misuse/poor implementation (75.7% vs. 59.2%, *p* = 0.02) and lack of clinical trial evidence (51.4% vs. 33.8%, *p* = 0.02) at significantly higher rates compared to APPs. No significant differences in concerns around MCD testing were observed by location or years in practice. However, PCCs at TMCs endorsed testing accuracy as a concern at higher rates compared to those within the HCS (61.7% vs. 51.4%), while PCCs from HCSs endorsed concerns about MCDs replacing routine screening at higher rates compared to those at TMC (54.1% vs. 43%).

### 3.3. Preferences in Learning About MCD

Per Table 4, most PCCs preferred to learn about MCDs via online courses and tutorials (64.5%), followed by classroom lectures and seminars (52.3%), guest lectures from industry (29.2%), shadowing MCD experts (23.4%), self-directed exploration (19.6%), and mentored research projects (14.1%). Significant differences in learning preferences were observed by location, degree, and years in practice. Specifically, those within the TMC were significantly more likely to prefer opportunities to shadow experts (29.4% vs. 13.8%, *p* = 0.04) and receive mentorship on research projects involving MCD (19.4% vs. 6.2%, *p* = 0.02). APPs preferred guest lectures from industry (37.9% vs. 24%, *p* = 0.03) and shadowing experts (32.3% vs. 18.2%, *p* < 0.01) at significantly higher rates compared to MD/DOs, and those with less than 10 years in their role endorsed guest lectures (34.9% vs. 25.7%, *p* = 0.04) and shadowing experts (34.4% vs. 16.5%, *p* < 0.01) at significantly higher rates compared to those with at least 10 years of experience.

## 4. Discussion

MCD tests have the potential to transform cancer screening and improve patient outcomes if implemented appropriately. This study adds to the limited evidence base around perspectives of PCCs towards MCD testing and provides critical insights into potential targets for future implementation efforts. Our findings align with prior studies showing that PCCs hold positive views towards MCD testing [22,23,24]. However, we observed gaps in knowledge and self-efficacy related to MCD testing and concerns around the cost and potential misuse/poor implementation with differences observed in location, degree, and years in practice. This study is one of the first to elicit preferences for learning about MCD testing, which mainly includes online courses and classroom instruction, though differences in learning preferences were observed by location, degree, and years in practice.

MCD testing may end up being performed alongside existing comprehensive screening efforts currently managed by PCCs in community clinical settings [25]. PCCs, including APPs, will need the knowledge and skills to initiate discussions around MCD testing, to order, interpret, communicate results and coordinate follow up care for those with a positive test. We observed low levels of knowledge and awareness around MCD testing overall with even lower levels observed among PCCs within the community-based healthcare system, APPs, and those with less than 10 years in practice. While these findings should be considered with caution due to small sample sizes, they could also reflect differences in exposure to enterprise initiatives. Findings from subgroup analyses found that PCCs working within the HCS sites were significantly more likely to be in family medicine (82.4% vs. 47.7%), while those working within the TMC sites were significantly more likely to be in internal medicine (47.7% vs. 13.5%). MCD testing is already underway at the TMC sites, with over 5000 patients having undergone testing. Physicians at the TMC sites have also been integral in developing pathways for diagnostic evaluation of positive MCD signals based upon iterative expert opinion and have made these available through electronic health record order sets and internal knowledge modules [26]. While we excluded individuals with prior experience with MCD testing, these efforts may explain differences observed by location. Differences observed by degree and years in practice are less clear but could reflect differences in experience and confidence with new technologies.

Understanding and addressing knowledge is foundational for designing effective implementation strategies to ensure successful adoption and sustained use of new clinical practices. In fact, PCCs in our study strongly preferred online courses followed by classroom seminars and instructions when learning about MCD testing. However, the evolving MCD landscape will create challenges for how best to educate PCCs, given differences in technologies, operating characteristics, and diagnostic algorithms, noting significant overlap between various tumors in regard to cell-free DNA methylation patterns. Notably, two of the leading MCD tests coming to the market (Galleri^®^ by GRAIL and CancerGuard™ by Exact Sciences) utilize different modalities of testing and different approaches to diagnostic evaluation [12,27]. The Galleri^®^ test analyzes cell-free DNA methylation patterns and provides a cancer signal origin to help direct diagnostic testing. In contrast, CancerGuard™ utilizes a combination of cell-free DNA methylation patterns, DNA mutations and serum protein biomarkers with only a positive or negative result, prompting clinical assessment for targeting diagnostic testing. As such, ongoing educational efforts for PCCs may be better focused on test performance, interpretation, and discussion surrounding the pros and cons of evolving MCD technologies to promote shared decision making [28,29,30].

PCCs in our study reported low levels of confidence/self-efficacy in their ability to interpret and communicate results and offer care based on MCD results, with APPs reporting significantly lower levels of confidence compared to MD/DOs. Additionally, 83% of PCCs were worried about managing patients with a positive result. These findings are in contrast to previous research, which found that primary care providers had high levels of confidence in their ability to manage and communicate results to patients [22]. Our findings may be attributed to restricting analyses to those with no direct experience with MCD testing. Even so, existing performance data suggest that a typical PCC ordering about 100 tests per year may only encounter one to two positive results [12,14]. This is hardly enough to garner confidence and expertise in managing diagnostic workflows, especially when first round testing fails to confirm a malignancy. PCCs may benefit from consulting with specialists when evaluating positive tests via dedicated MCD clinics [20,26]. These clinics can aid in evaluating patients with positive MCD signals and help address the challenges facing overstretched PCCs by providing expert, specialized care to patients through vetted diagnostic evaluations and individualized surveillance plans. This is an approach that our healthcare system is pursuing as we roll out MCD testing to a broader group of PCCs and may lead to greater adoption of MCD testing and improved outcomes. However, the capacity required to establish and staff dedicated MCD clinics will vary across healthcare settings. For instance, safety net settings like Federally Qualified Health Centers may lack the capacity to accommodate the diagnostic processes associated with a positive MCD result. To this end, meaningful partnerships between community-based clinics and tertiary/academic medical centers with the capacity for MCD are needed [31]. Additionally, navigational services can also help individuals overcome barriers to care, particularly when diagnostic testing and treatment are required following MCD testing [32].

In line with prior studies [22,23,24], PCCs in our study endorsed cost, misuse/poor implementation, and accuracy of MCD tests as concerns to MCD testing. Prior studies with clinicians and other key stakeholders, including patients, indicate cost as a key challenge that can undermine patients’ ability to access testing and follow-up care and a health system’s ability to offer MCD testing [10,24,29,33]. Some MCD tests, like Galleri^®^ [34] and CancerGuard™, are commercially available as laboratory-developed tests for an out-of-pocket cost that is largely out of reach for many patients. While legislation is underway to provide coverage for MCD tests upon FDA approval [35], coverage for diagnostic follow up remains unclear, as well as overall costs to healthcare systems associated with MCD implementation. As current trials focus on the clinical safety and utility of MCD testing, serious implementation questions remain, including workflow processes, procedures, and unintended consequences (i.e., misuse and harm) [36,37]. Various organizations, including the Multicancer Earyl Detection Consortium, are in the process of developing recommendations for implementation, including standardized workflows and procedures for use of MCDs in clinical settings and processes to promote shared decision making between providers and patients [8,28,29,31,32,38]. While important, the ability for PCCs, particularly those in community clinics, to adopt these efforts may vary due to resource and time constraints. Electronic health record systems, clinical decision support tools, decision aids, and results interpretation assistance have been proposed as strategies to minimize workforce burden but are largely targeted towards point of care MCD testing rather than the entire clinical pathway (i.e., diagnostic follow-up) [24,30]. This is particularly concerning for low resourced and/or community clinical settings that lack the resources and expertise to support those with a positive test [32]. To this end, more research is needed to understand how best to implement MCD testing in a safe and ethical manner that aligns with existing workflows, processes, and capacity.

There are several limitations to this study. Although our study includes PCCs spanning a large, geographically diverse healthcare enterprise, this limits our ability to generalize to other settings. While our study explored differences in perspectives through important characteristics, these findings should be considered with caution due to our low response rate and small sample sizes limiting our analyses to descriptive summaries. The analysis was limited to those with no prior experience with MCD testing and survey items assessed anticipated concerns, knowledge, attitudes, and self-efficacy towards key aspects of MCD testing. While important for gaining initial insights into potential barriers and needs around MCD testing, response options were limited to pre-determined options and likely do not represent all challenges and concerns associated with MCD testing. Additionally, we included perspectives from internal medicine PCCs, many working within a well-resourced TMC, where perspectives around MCD testing implementation likely differ compared to PCCs from a community-based primary care clinics. Future studies with larger, more representative samples are needed to identify appropriate targets for future implementation efforts.

## 5. Conclusions

MCD testing presents a unique opportunity to plan for implementation throughout the evidence-generation process. Our study is unique as we gathered perspectives from PCCs, including APPs, from a large, geographically diverse healthcare enterprise enabling examination of differences by individual and system-level factors. Moreover, this is one of the first studies to elicit preferences for learning about MCD testing. In summary, we found that PCCs in our study held positive views towards MCD testing. However, gaps and variation in knowledge, awareness, and self-efficacy towards MCD testing by location, degree, and years of practice emphasize the need for tailored implementation strategies. While efforts to train and educate all PCCs on MCD testing is a critical first step, more research is needed to understand how best to support implementation across the MCD testing continuum. This includes understanding and developing strategies that support gaps in current capacity, workflows, and processes beginning at point of care testing through diagnostic follow-up testing to the communication of results, and accounting for differences across PCCs and clinical settings.

## Figures and Tables

**Table 1:** Summary of survey respondents (*N* = 181).

	Overall
Location	
TMC	107 (59.2)
HCS	74 (40.8)
Role	
MD/DO	107 (59.1)
APP	71 (39.2)
Other ^1^	2 (1.1)
Departments	
Internal Medicine	61 (33.7)
Family Medicine	112 (61.9)
Other ^2^	8 (4.4)
Years in Practice	
<10 years	67 (37)
≥10 years	114 (63)

Abbreviations: TMC—Tertiary medical center; HCS—Healthcare system; APP—Advanced practice provider. ^1^ Includes resident/fellow, other. ^2^ Includes women’s health, international, other.

**Table 2:** Overall knowledge, attitudes, and self-efficacy towards MCD testing.

	*N* (% Agree)
Knowledge/Awareness	
I am familiar with the basic concepts of MCD in healthcare.	69 (39.0)
I understand how MCD testing works.	62 (35.0)
Attitudes	
MCD tests will take on a significant role in healthcare.	122 (67.8)
I am interested in using MCD tests in my practice.	120 (66.3)
I am concerned about having the time to explain the test to my patients.	141 (77.9)
I am worried about managing my patients with a positive blood test result.	146 (80.7)
Self-Efficacy	
I feel confident in my ability to offer/provide direct care for my patients based on MCD results.	41 (22.9)
I feel confident in my ability to interpret and communicate MCD results to the patient.	30 (16.9)
I am comfortable understanding the limitations of MCD tests.	73 (41.2)

**Table 3:** Knowledge, attitudes, and self-efficacy towards MCD testing by location, degree, and years in practice.

	TMC (*N* = 107)	HCS (*N* = 74)	*p* Value	APP (*N* = 71)	MD/DO (*N* = 107)	*p* Value	<10 Years (*N* = 67)	≥10 Years (*N* = 114)	*p* Value
**Knowledge/Awareness**									
I am familiar with the basic concepts of MCD in healthcare.			<0.01			<0.01			0.04
Agree	51 (48.6%)	18 (25.0%)		14 (20.0%)	53 (51.0%)		19 (29.2%)	50 (44.6%)	
Neutral/Disagree	54 (51.4%)	54 (75.0%)		56 (80.0%)	51 (49.0%)		46 (70.8%)	62 (55.4%)	
I understand how MCD testing works.			<0.01			<0.01			0.01
Agree	46 (43.8%)	16 (22.2%)		11 (15.7%)	49 (47.1%)		15 (23.1%)	47 (42.0%)	
Neutral/Disagree	59 (56.2%)	56 (77.8%)		59 (84.3%)	55 (52.9%)		50 (76.9%)	65 (58.0%)	
**Attitudes**									
MCD tests will take on a significant role in healthcare.			0.31			0.52			0.26
Agree	75 (70.8%)	47 (63.5%)		47 (66.2%)	75 (70.8%)		42 (62.7%)	80 (70.8%)	
Neutral/Disagree	31 (29.2%)	27 (36.5%)		24 (33.8%)	31 (29.2%)		25 (37.3%)	33 (29.2%)	
I am interested in using MCD tests in my practice.			0.19			0.98			0.61
Agree	75 (70.1%)	45 (60.8%)		47 (66.2%)	71 (66.4%)		46 (68.7%)	74 (64.9%)	
Neutral/Disagree	32 (29.9%)	29 (39.2%)		24 (33.8%)	36 (33.6%)		21 (31.3%)	40 (35.1%)	
I am concerned about having the time to explain the test to my patients.			0.90			0.56			0.94
Agree	83 (77.6%)	58 (78.4%)		57 (80.3%)	82 (76.6%)		52 (77.6%)	89 (78.1%)	
Neutral/Disagree	24 (22.4%)	16 (21.6%)		14 (19.7%)	25 (23.4%)		15 (22.4%)	25 (21.9%)	
I am worried about managing my patients with a positive blood test result.			0.30			0.83			0.45
Agree	89 (83.2%)	57 (77.0%)		58 (81.7%)	86 (80.4%)		56 (83.6%)	90 (78.9%)	
Neutral/Disagree	18 (16.8%)	17 (23.0%)		13 (18.3%)	21 (19.6%)		11 (16.4%)	24 (21.1%)	
**Self-Efficacy**				56 (80.0%)	51 (49.0%)				
I feel confident in my ability to offer/provide direct care for my patients based on MCD results.			0.53			0.12			0.97
Agree	26 (24.5%)	15 (20.5%)		12 (17.1%)	29 (27.4%)		15 (22.7%)	26 (23.0%)	
Neutral/Disagree	80 (75.5%)	58 (79.5%)		58 (82.9%)	77 (72.6%)		51 (77.3%)	87 (77.0%)	
I feel confident in my ability to interpret and communicate MCD results to the patient.			0.09			<0.01			0.67
Agree	22 (21.0%)	8 (11.1%)		5 (7.1%)	25 (24.0%)		10 (15.4%)	20 (17.9%)	
Neutral/Disagree	83 (79.0%)	64 (88.9%)		65 (92.9%)	79 (76.0%)		55 (84.6%)	92 (82.1%)	
I am comfortable understanding the limitations of MCD tests.			0.21			<0.01			0.77
Agree	29 (27.6%)	14 (19.4%)		10 (14.3%)	33 (31.7%)		15 (23.1%)	28 (25.0%)	
Neutral/Disagree	76 (72.4%)	58 (80.6%)		60 (85.7%)	71 (68.3%)		50 (76.9%)	84 (75.0%)	

**Table 4:** Learning Preferences Overall and by location, degree, and years in practice.

	Overall	TMC (*N* = 107)	HCS (*N* = 74)	*p* Value	APP (*N* = 71)	MD/DO (*N* = 107)	*p* Value	<10 Years (*N* = 67)	≥10 Years (*N* = 114)	*p* Value
Online courses and tutorials				0.87			0.66			0.20
Prefer a great deal/a lot	111 (64.5%)	66 (63.5%)	45 (66.2%)		47 (69.1%)	63 (62.4%)		46 (70.8%)	65 (60.7%)	
Prefer a moderate amount	39 (22.7%)	25 (24.0%)	14 (20.6%)		13 (19.1%)	24 (23.8%)		10 (15.4%)	29 (27.1%)	
Prefer slightly/Do not prefer	22 (12.8%)	13 (12.5%)	9 (13.2%)		8 (11.8%)	14 (13.9%)		9 (13.8%)	13 (12.1%)	
Classroom lectures and seminars				0.42			0.34			0.81
Prefer a great deal/a lot	90 (52.3%)	55 (53.4%)	35 (50.7%)		36 (52.9%)	53 (52.5%)		35 (53.8%)	55 (51.4%)	
Prefer a moderate amount	43 (25.0%)	28 (27.2%)	15 (21.7%)		20 (29.4%)	22 (21.8%)		17 (26.2%)	26 (24.3%)	
Prefer slightly/Do not prefer	39 (22.7%)	20 (19.4%)	19 (27.5%)		12 (17.6%)	26 (25.7%)		13 (20.0%)	26 (24.3%)	
Mentored research projects				0.02			0.89			0.23
Prefer a great deal/a lot	23 (14.1%)	19 (19.4%)	4 (6.2%)		10 (15.6%)	13 (13.4%)		10 (16.1%)	13 (12.9%)	
Prefer a moderate amount	23 (14.1%)	16 (16.3%)	7 (10.8%)		8 (12.5%)	14 (14.4%)		12 (19.4%)	11 (10.9%)	
Prefer slightly/Do not prefer	117 (71.8%)	63 (64.3%)	54 (83.1%)		46 (71.9%)	70 (72.2%)		40 (64.5%)	77 (76.2%)	
Guest lectures from industry experts				0.06			0.03			0.04
Prefer a great deal/a lot	49 (29.2%)	36 (35.3%)	13 (19.7%)		25 (37.9%)	24 (24.0%)		22 (34.9%)	27 (25.7%)	
Prefer a moderate amount	37 (22.0%)	18 (17.6%)	19 (28.8%)		17 (25.8%)	19 (19.0%)		18 (28.6%)	19 (18.1%)	
Prefer slightly/Do not prefer	82 (48.8%)	48 (47.1%)	34 (51.5%)		24 (36.4%)	57 (57.0%)		23 (36.5%)	59 (56.2%)	
Shadowing MCD experts				0.04			<0.01			<0.01
Prefer a great deal/a lot	39 (23.4%)	30 (29.4%)	9 (13.8%)		21 (32.3%)	18 (18.2%)		22 (34.4%)	17 (16.5%)	
Prefer a moderate amount	27 (16.2%)	13 (12.7%)	14 (21.5%)		16 (24.6%)	10 (10.1%)		15 (23.4%)	12 (11.7%)	
Prefer slightly/Do not prefer	101 (60.5%)	59 (57.8%)	42 (64.6%)		28 (43.1%)	71 (71.7%)		27 (42.2%)	74 (71.8%)	
Self-directed exploration and experimentation with MCD testing				0.06			0.18			0.69
Prefer a great deal/a lot	33 (19.6%)	26 (25.5%)	7 (10.6%)		8 (12.5%)	24 (23.8%)		11 (17.2%)	22 (21.2%)	
Prefer a moderate amount	32 (19.0%)	19 (18.6%)	13 (19.7%)		12 (18.8%)	19 (18.8%)		14 (21.9%)	18 (17.3%)	
Prefer slightly/Do not prefer	103 (61.3%)	57 (55.9%)	46 (69.7%)		44 (68.8%)	58 (57.4%)		39 (60.9%)	64 (61.5%)	

## Data Availability

Summary level data and datasets used and/or analyzed without individual data are available from the corresponding author with permission.

## Supplementary Materials

The following supporting information can be downloaded at: https://www.mdpi.com/article/10.3390/jpm15100452/s1. File S1: Survey.

## Abbreviations

The following abbreviations are used in this manuscript:

MCD	Multicancer detection testing
PCC	Primary care clinician
APP	Advanced Practice Provider
NP	Nurse Practitioner
PA	Physician Assistant
HCS	Healthcare system
TMC	Tertiary academic medical center
FDA	Food and Drug Administration
