# Supplementary material for: Primary Care Clinician Preferences and Perspectives on Multi-Cancer Detection Testing Across an Integrated Healthcare System
Source: J Pers Med. 2025 Sep 28;15(10):452. doi: 10.3390/jpm15100452 (PMC12565203; doi:10.3390/jpm15100452)
Supplement: Supplementary file 1 [file jpm-15-00452-s001.zip › jpm-3842178-supplementary.pdf]

MCD Testing Survey

Objectives: Our goal is to enhance the understanding and awareness of multi-cancer detection (MCD) tests among Mayo Clinic clinicians, aiming to support providers in incorporating these tests into their practice effectively. Additionally, we seek to identify and fulfill the educational needs of providers regarding MCD tests to ensure they feel confident and interested in utilizing this innovative testing approach for the benefit of patient care.

Introduction: Novel, high-performance genomic technologies now allow detection of signals from cancers in the blood, giving rise to a new paradigm of multi-cancer detection (MCD). Blood is inherently well suited for detection of cancer biomarkers as it contains circulating tumor cells and tumor cell-free DNA (cfDNA) that have been "shed" from an occult asymptomatic developing cancer. An MCD test would analyze these genomic features of the circulating DNA, distinguish it from background signals and determine a site of tumor origin to guide subsequent testing to establish a firm cancer diagnosis (hopefully pre-symptomatic early stage and highly curable).

Intent: The data that is collected through this survey will allow our team to develop and deliver a new service line offering based around Multi-Cancer Detection.

Thank you for filling out this survey and for your time. This survey is completely voluntary.

About You

Q1 Please provide your location:

- ○ Arizona (1)
- ○ Florida (2)
- ○ Rochester (3)
- ○ Mayo Clinic Health System (4)

Q2 What department/division are you affiliated with?

- General Internal Medicine (1)
- Community Internal Medicine (2)
- Family Medicine (3)
- Women's Health (4)
- International (5)
- Other (6) ____

Q3 What is your current role?

- Resident/Fellow (1)
- NP/PA (2)
- Physician (3)
- Other Clinician (4) ____

Q4 How long have you been in practice?

- < 1 year (1)
- 1-5 years (2)
- 5-10 years (3)
- > 10 years (4)

Experience & Thoughts about Multi-Cancer Detection (MCD) Tests

Q5 Have you had any experience ordering MCD tests in your practice?

- ◯ Yes (1)
- ◯ No (2)

Q6 I am interested in using MCD tests in my practice.

- ◯ Strongly Agree (1)
- ◯ Somewhat agree (2)
- ◯ Neither agree nor disagree (3)
- ◯ Somewhat disagree (4)
- ◯ Strongly disagree (5)

Q7 Do you have any concerns about the use of MCD tests in healthcare? (check all that apply)

- ☐ Concerns of misuse and poor implementation/interpretation (1)
- ☐ Concern that patients will replace routine screening (i.e. Mammogram, colonoscopy, etc.) with MCD test (2)
- ☐ Concerns about the lack of definitive clinical trial evidence (3)
- ☐ Concerns about the lack of FDA approval of this test (4)
- ☐ Accuracy of the test (5)
- ☐ Cost of the test (6)
- ☐ Other (7) ____
- ☐ I do not have any concerns (8)

Q8 I am concerned about having the time to explain the test to my patients.

- ○ Strongly Agree (1)
- ○ Somewhat agree (2)
- ○ Neither agree nor disagree (3)
- ○ Somewhat disagree (4)
- ○ Strongly disagree (5)

Q9 I am worried about managing my patients with a positive blood test result.

- ◯ Strongly Agree (1)
- ◯ Somewhat agree (2)
- ◯ Neither agree nor disagree (3)
- ◯ Somewhat disagree (4)
- ◯ Strongly disagree (5)

Q10 I feel confident in my ability to offer/provide direct care for my patients based on MCD results.

- ◯ Strongly Agree (1)
- ◯ Somewhat agree (2)
- ◯ Neither agree nor disagree (3)
- ◯ Somewhat disagree (4)
- ◯ Strongly disagree (5)

Q11 Having a dedicated clinic for positive blood test results would be desirable.

- ◯ Strongly agree (9)
- ◯ Somewhat agree (10)
- ◯ Neither agree nor disagree (11)
- ◯ Somewhat disagree (12)
- ◯ Strongly disagree (13)

Q12 MCD tests will take on a significant role in healthcare.

- ○ Strongly agree (1)
- ○ Somewhat agree (2)
- ○ Neither agree nor disagree (3)
- ○ Somewhat disagree (4)
- ○ Strongly disagree (5)

Q13 Which of the following areas do you think should be prioritized for MCD implementation?

Please rank each area and place them in order from 1 to 5, with 1 being the highest priority and 5 being the lowest priority.

_______ General Internal Medicine (1)
_______ Community Internal Medicine (2)
_______ Family Medicine (3)
_______ Women's Health (4)
_______ International (5)
_______ Medallion (6)

Understanding and Education/Training with MCD Tests

Q14 I am familiar with the basic concepts of MCD in healthcare.

- ◯ Strongly agree (1)
- ◯ Somewhat agree (2)
- ◯ Neither agree nor disagree (3)
- ◯ Somewhat disagree (4)
- ◯ Strongly disagree (5)

Q15 I understand how MCD testing works.

- ◯ Strongly agree (1)
- ◯ Somewhat agree (2)
- ◯ Neither agree nor disagree (3)
- ◯ Somewhat disagree (4)
- ◯ Strongly disagree (5)

Q16 I feel confident in my ability to interpret and communicate MCD results to the patient.

- ◯ Strongly agree (1)
- ◯ Somewhat agree (2)
- ◯ Neither agree nor disagree (3)
- ◯ Somewhat disagree (4)
- ◯ Strongly disagree (5)

Q17 I am comfortable understanding the limitations of MCD tests.

- Strongly agree (1)
- Somewhat agree (2)
- Neither agree nor disagree (3)
- Somewhat disagree (4)
- Strongly disagree (5)

Q18 Have you had any formal or informal education on MCD tests?

- Yes (1)
- No (2)

Q19 Have you engaged in any of the following activities? (Select all that apply)

- Attendance at an MCD course/WebX (1)
- MCD based talk at a conference (2)
- Reading MCD based research articles/journals/blogs (3)
- Involvement in MCD project (4)
- No involvement in MCD based activities (5)
- No interest in MCD based projects (6)

Q20 In your opinion, what are the ways in which you would prefer to learn MCD testing during training?

Please rate each method on a preference scale.

	Prefer a great deal (1)	Prefer a lot (2)	Prefer a moderate amount (3)	Prefer slightly (4)	Do not prefer (5)
Online courses and tutorials (1)	○	○	○	○	○
Classroom lectures and seminars (2)	○	○	○	○	○
Mentored research projects involving MCD in healthcare (3)	○	○	○	○	○
Guest lectures from industry MCD experts (4)	○	○	○	○	○
Shadowing MCD experts in healthcare settings (5)	○	○	○	○	○
Self-directed exploration and experimentation with MCD testing (6)	○	○	○	○	○
Other (7)	○	○	○	○	○

Q21 Are you interested in becoming more involved in MCD testing? If yes, please provide your email.

○ Yes (4) ____

○ No (5)
